# Mapping the Volume Transfer of Graphene-Based Inks with the Gravure Printing Process: Influence of Rheology and Printing Parameters

**DOI:** 10.3390/ma15072580

**Published:** 2022-03-31

**Authors:** Ahmad Fakhari, Célio Fernandes, Francisco José Galindo-Rosales

**Affiliations:** 1 Transport Phenomena Research Center (CEFT), Mechanical Engineering Department, Faculty of Engineering of the University of Porto, Rua Dr. Roberto Frias s/n, 4200-465 Porto, Portugal; ahmadfakhari@fe.up.pt; 2 ALiCE-Associate Laboratory in Chemical Engineering, Faculty of Engineering, University of Porto, Rua Dr. Roberto Frias, 4200-465 Porto, Portugal; 3 LASI-Associate Laboratory of Intelligent Systems, Institute for Polymers and Composites, Polymer Engineering Department, School of Engineering of the University of Minho, Campus of Azurém, 4800-058 Guimarães, Portugal; cbpf@dep.uminho.pt; 4 Transport Phenomena Research Center (CEFT), Chemical Engineering Department, Faculty of Engineering of the University of Porto, Rua Dr. Roberto Frias s/n, 4200-465 Porto, Portugal

**Keywords:** graphene-based inks, rheology, gravure printing process, OpenFoam, RheoTool

## Abstract

It is a common practice to add rheology modifiers to functional inks, such as graphene inks, to optimize the rheological properties so that they can be printed with a certain printing technique. This practice may lead to inks formulations with poorer electrical, optical, and mechanical performance upon its application, which are of paramount importance in printed electronics. In this study, we demonstrate for three different commercial graphene-based inks that it is possible to control the amount of ink transferred to the flat surface by tweaking printing parameters, such as the velocity and the length scale of the gravure cell, without modifying the rheology of the ink. Finally, the results are summarized in printing maps based on dimensionless numbers, namely, the capillary and Reynolds numbers.

## 1. Introduction

Printed electronics refers to the application of printing techniques to fabricate electronic structures, such as devices and circuits, by processing a functional material (ink) from the liquid phase onto a substrate. Graphene-based inks are functional inks consisting of a dispersion of graphene nanoparticles in a carrier liquid. A good dispersion of graphene provides the basis for ensuring the excellent electrical, mechanical, thermal, and optical properties of graphene-based inks, and these properties are the reason why these inks represent the holy grail for printed flexible electronics [1,2]. However, a mass production method for high-quality graphene free from any defect is a challenging task, and, while the direct exfoliation of pristine graphene in liquid media has shown promising results, the development of conductive inks still faces problems of paramount importance from the application point of view, such as unsuitable viscosity [3]. In order to improve their printability, it is a common practice to introduce rheology modifiers so that the graphene inks can be properly printed with state-of-the-art printing techniques. Additives may correct the rheological properties of the graphene-based inks up to a certain level, but this improvement is to the detriment of the stability and finesse of the combination between the electrical-mechanical-thermal-optical properties required for the final product [4]. This current approach of trying to tune the rheological behavior without losing the performance of the final 2D ink is like fitting a round peg in a square hole. It seems rather more reasonable to formulate the graphene ink keeping in mind just its stability, the adhesion with the substrate, and the final mechanical and electrical properties of the conductive features; then, based on the rheological properties of the resulting ink, one should select the right printing process and tweak the printing parameters according to the fluidic properties of the ink in order to optimize the printability of the functional ink.

The portfolio of fabrication techniques for printed electronics comprises a wide range of methods, including screen [5], gravure [6], flexographic [7], inkjet [8], aerosol jet printing [9], and electrohydrodynamic jet (e-jet) printing [10], which have been widely used for the fabrication of thin-film transistors, radiofrequency identifications, displays, etc. Among the plethora of printing techniques, gravure printing is ideally suited to printed electronics because it offers a high throughput [11] to allow mass production and, at the same time, it offers the possibility of producing high-quality patterns at a micron-scale resolution [12]. However, in spite of several research groups having demonstrated gravure-printed features larger than 10 μm [13], few works have gone down to the 1 μm range [14], and, to the best of authors’ knowledge, this length scale has never been reported for graphene inks [3].

In order to reach that resolution in patterning with graphene inks by means of the gravure printing process, it is of paramount importance to understand the role that the rheology of the ink plays in the physics underneath each of the four main steps involved: the first step is the *filling* in which the gravure cells are overfilled by the ink; the second step is the *wiping*, where a doctor blade removes the excess ink; the third step is the *transferring*, where the substrate contacts the ink contained in the gravure cells and then the ink is picked out and transferred to the substrate; and the last step is the *levelling*, in which the individual droplets deposited onto the substrate are spread by gravity to create a continuous pattern [15]. Very recently, Wu et al. [16] numerically analyzed the effect of shear thinning on the liquid filling problem involving a stationary trapezoidal cavity and a horizontal plate above the cavity. Kitsomboonloha et al. [14] could not experimentally observe any effect of the non-Newtonian character of the ink on the wiping process, and they claimed that it was the dimensionless capillary number ($Ca=\frac{\eta v}{\sigma }$) that was controlling this process so that, for the same viscosity (η) and velocity (*v*), using doctor blades with a lower surface tension (σ) results in smaller drag-out tails; however, Wu et al. [17] demonstrated numerically that a shear-thinning behavior improves cavity emptying compared to the Newtonian behavior, whereas the opposite was observed for shear-thickening liquids. In the transferring process for Newtonian fluids [18,19], it is the capillary number controlling the amount of fluid transferred; nevertheless, it has been experimentally proven that viscoelasticity [20,21] and shear thickening [22] increases the amount of fluid transferred. Rothstein and co-workers [20,21,22] showed that the non-Newtonian character of the inks strongly affects the liquid transfer during gravure printing. It is important to note that their experimental studies were performed with relatively large gravure cells, and wetting/de-wetting may be a critical phenomenon in determining pick out at low stretch rates under gravitational forces. The final levelling of the ink depends strongly on its rheological properties; thus, a Newtonian-like ink would tend to achieve a highly uniform film over large areas, while a pseudoplastic behavior would allow for highly scaled features [15].

In this study, we aim at clarifying the role of the shear-thinning behavior of graphene inks in the transferring process of gravure printing separately when the length scale of the gravure cell scales down from millimetres to the order of microns. To do so, we modeled the rheological behavior of three commercial graphene-based inks with generalized Newtonian fluid (GNF) models, and we performed 2D axisymmetric numerical simulations in the open-source computational library OpenFOAM for several gravure cell sizes and printing velocities. The simulations were validated by reproducing the experimental results reported in Sankaran and Rothstein [20] and with our own experimental results obtained under simple shear and uniaxial extensional flows.

This work is organized as follows: in Section 2, the commercial inks, their rheological characterization, and the constitutive models are detailed; then, the fluid domain and the governing equations as well as the boundary and initial conditions are presented in Section 3, together with the numerical method used for the solution of this system of differential equations and the mesh used and its validation; in Section 4, we present the results and discussion; and we conclude with a summary and remarks on the work (Section 5).

## 2. Rheology of the Graphene-Based Inks

### 2.1. Graphene Inks

**Figure 1:**
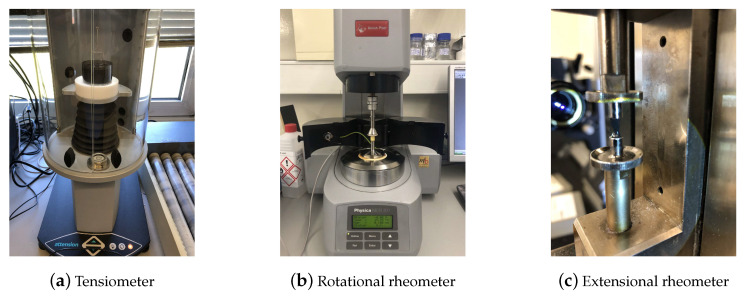
Experimental devices used for the measurement of the surface tension, i.e., (**a**) a Sigma 700 Biolin Scientific equipped with a Du Noüy ring, and the rheological characterization of the commercial graphene inks, i.e., (**b**) an Anton Paar MCR301 and (**c**) a CaBER-1.

Three commercial graphene inks from Graphenest S.A. (Aveiro, Portugal) were used [23]. In all cases, the solid phase consisted of graphene nanoparticles dispersed in a carrier fluid. The properties provided by the manufacturer are shown in Table 1, together with the surface tension, which was measured at ∼20 °C utilizing a force tensiometer (Sigma 700 Biolin Scientific, Espoo, Finland) equipped with a Du Noüy ring of 0.185 mm in thickness and 9.58 mm in diameter (Figure 1a). As the rheological properties of colloidal suspensions may depend on the dispersion quality [24], fresh samples were always used for each measurement after being re-dispersed according to the manufacturer instructions. It is also known that the size of graphene flakes will have an influence on the rheological properties of the graphene-based inks; the three commercial inks presented a wide distribution of particle sizes (1 μm to 20 μm), and the flakes’ thickness was below 10 nm. For more information, we encourage the reader to visit the website of the manufacturer [23].

### 2.2. Shear Rheometry

The rheological characterization under simple shear flow was performed by means of a stress-controlled rotational rheometer (Anton Paar MCR301, Graz, Austria) using parallel plates of 50 mm (*D*) and a 0.1 mm gap (*h*) (Figure 1b). All the experiments were performed at 20 °C and used a solvent trap to avoid partial evaporation of the carrier fluid of the samples. The steady shear viscosity curves were obtained between 1 and 10,000 s${}^{-1}$.

It is well known that colloidal suspensions, and particularly inks, exhibit preshear history dependence [25]. Preshearing the sample before running the rheological experiment is widely recognized as a necessary step to guarantee repeatability in rheological studies of these materials. Therefore, in order to ensure the results were reproducible, before each experiment, a steady preshear rate was applied up to an equilibrium state, followed by a rest state. We followed the same criteria defined by K. Dullaert [26] and successfully replicated by Galindo-Rosales et al. [27]. In this way, any previous shear history on the sample was erased [28]. The value of the shear rate applied in this preshear stage is the limit of reversibility of the sample in order to ensure that they have not been denatured. Afterwards, the samples were kept at rest for a period of time that was long enough to let them achieve an equilibrium structure and temperature. Thus, the protocol for the rheological experiments in simple shear flows consisted of three steps: (1) the preshear, (2) the rest period, and (3) the rheological test itself. Table 2 summarizes the preshear rates, the time of preshearing, and the rest time provided for each sample.

### 2.3. Extensional Rheometry

The rheological characterization under extensional flow was performed in the Capillary Break-up Extensional Rheometer (HAAKE CaBER 1, Thermo Fisher Scientific, Waltham, MA, USA) equipped with 4 mm diameter plates ($D_{0}$) as shown in Figure 1c. The temperature control was set to 20 °C (Thermo Haake DC10, Karlsruhe, Germany).

The experimental procedure was set based on the information obtained from the shear rheometry and the surface tension measurements. As the three samples exhibited a viscosity well above the minimum value required for successful measurements of the capillary thinning process without the influence of inertial effects [29], the slow retraction method was not applied this time [30]. The sample was loaded between the parallel plates and the initial height was set at $h_{0}=\frac{D_{0}}{2}$; then, a step-wise function was applied to the top plate so that the final height of $h_{f}=5.6$ mm was reached in 20 ms following a linear stretching profile. Thus, the three samples were subjected to a Hencky strain of ϵ∼1.05.

The silhouette of the filament thinning process was recorded by means of a high-speed camera (Photron FASTCAM Mini UX100, West Wycombe, Buckinghamshire, UK) equipped with a set of optical lenses (Optem Zoom 70 XL, Qioptiq, Fairport, NY, USA); to establish the correct illumination and achieve high a contrast in the images of the liquid bridge profile, a 52 mm telecentric backlight illuminator was used, connected to a metal halide light source (LeicaEL6000, Leica Microsystems, Wetzlar, Germany) by an optical fibre cable, as detailed in recent works [31,32,33,34,35]. The optical setup was previously calibrated, providing resolutions of 6.8 μm/pix. The time evolution of the filament thinning process was recorded from the beginning of the experiment at 8000 fps and post-processed in Matlab, following the same procedure as described in our recent works [31,33,34,36].

## 3. Geometry, Equations and Numerical Method

### 3.1. Geometry, Boundary, and Initial Conditions

**Figure 2:**
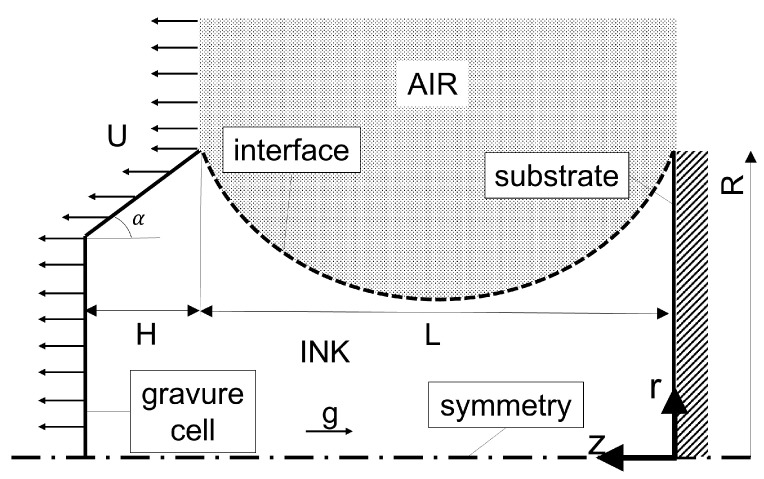
Sketch of the domain employed for the gravure cell printing process. The main dimensional parameters are: R=2.5mm, H=1mm, α=15${}^{{}^{\circ}}$, and $L_{0}\leq L\leq L_{b}$, where $L_{0}=0.75\;$mm and $L_{b}$ is the breakup length.

In this study, we intend to numerically analyze how the shear thinning behavior and printing parameters, namely, the velocity and size of the gravure cell, affect the amount of fluid transferred from the gravure cell to the substrate for three different graphene-based inks. To emulate the printing process, we considered a simplified 2D axisymmetric version of the gravure cell, as depicted in Figure 2, similar to the one proposed by Sankaran and Rothstein [20]. The ink is initially at rest, contained between the gravure cell and the substrate walls, which are separated by a distance, $L_{0}$. The liquid bridge is surrounded by air. The substrate is fixed, while the gravure cell moves away from the substrate at a constant speed, *U*. Therefore, the touchdown and the shearing process that would occur in the real roll-to-roll coating are not considered in this study. We focus on the analysis of the separation process and the transference of fluid to the substrate for each case.

Up to four different lengthscales have been considered in this study, i.e., the same set of dimensions shown in Figure 2 have been equally scaled down by factors of 1/10, 1/100, and 1/1000. Thus, for instance, the characteristic radius for each set of simulations was $R=\left\{0.0025,0.025,0.25,2.5\right\}\;$mm.

Figure 2 displays the computational domain and the boundaries. A no-slip boundary condition with zero velocity at the substrate is imposed. At the gravure cell surface, there is a no-slip boundary condition, but the wall moves with a defined velocity, given by U=cte. At the top boundary, a zero velocity gradient is set. The two lateral side boundaries, front and back, are considered to be wedge patches, which means the cylinder is specified as a wedge of small angle, e.g., 5° and one cell thick, running along the plane of symmetry, straddling one of the coordinate planes. At the bottom, the axis of symmetry is considered to be an empty patch. A fixed uniform value of zero is imposed for the pressure at the top boundary where there is air, and, at the walls, zero gradient pressure is set. The volume fraction of the liquid at the walls is considered to be 1 where there is liquid and 0 where there is air.

### 3.2. Governing Equations

It is assumed that the flow is two-dimensional, axisymmetric, incompressible (ρ=cte), laminar, isothermal, and transient. The governing equations for such flow conditions are the mass conservation equation (Equation (1)):

(1) ∇·u=0,

and the momentum balance equation (Equation (2)):

(2) $$\rho \frac{Du}{Dt}=-\nabla p+\nabla \cdot \tau +\rho g+f_{s},$$

where ρ is the liquid density; $\frac{Du}{Dt}$ is the total time derivative of u, which is the velocity vector; *p* is the pressure; g is the acceleration of gravity (Figure 2); τ is the extra stress tensor, which is calculated using a constitutive equation (Section 3.2.1); and $f_{s}$ is the surface tension force at the liquid-air interface (Section 3.2.2).

#### 3.2.1. Working Fluids and Constitutive Models

The working fluids, i.e., the graphene inks, are surrounded by air (with a kinematic viscosity of $\nu =1.5\cdot 10^{-5}$ m${}^{2}$/s and a density of ρ=1.225 kg/m${}^{3}$). The surface tension between ink and air and the rheological properties of the inks were experimentally determined according to Section 2. In this study, we will analyze the effects of shear thinning and length scale in the volume of ink transferred from the gravure cell to the substrate. For that purpose, we compare the results obtained from appropriate GNF models. The selection of the GNF constitutive models for each ink is discussed below.

Generalized Newtonian fluid constitutive equations were developed from the Newtonian constitutive equation (constant viscosity) for incompressible fluids and represent the simplest constitutive models for non-Newtonian fluids (Equation (3)):

(3) $$\tau =\eta \left(\dot{\gamma }\right)\dot{\gamma },$$

where $\dot{\gamma }=\nabla u+\nabla u^{T}$ is the shear rate tensor, $\dot{\gamma }=\sqrt{\frac{\dot{\gamma }:\dot{\gamma }}{2}}$ is the magnitude of the shear rate tensor, and $\eta \left(\dot{\gamma }\right)$ is the dynamic viscosity as a function of the shear rate [37]. Consequently, GNF models can only account for variable viscosity effects through the function $\eta \left(\dot{\gamma }\right)$ but not for normal stress differences ($N_{1}=\tau_{11}-\tau_{22}=0$ and $N_{2}=\tau_{22}-\tau_{33}=0$) [38], and, for that reason, they are considered to be inelastic models. The GNF models are quite general since the functional form of $\eta \left(\dot{\gamma }\right)$ must fit to data in order for flow properties to be predicted, and that function is typically empirical.

**Figure 3:**
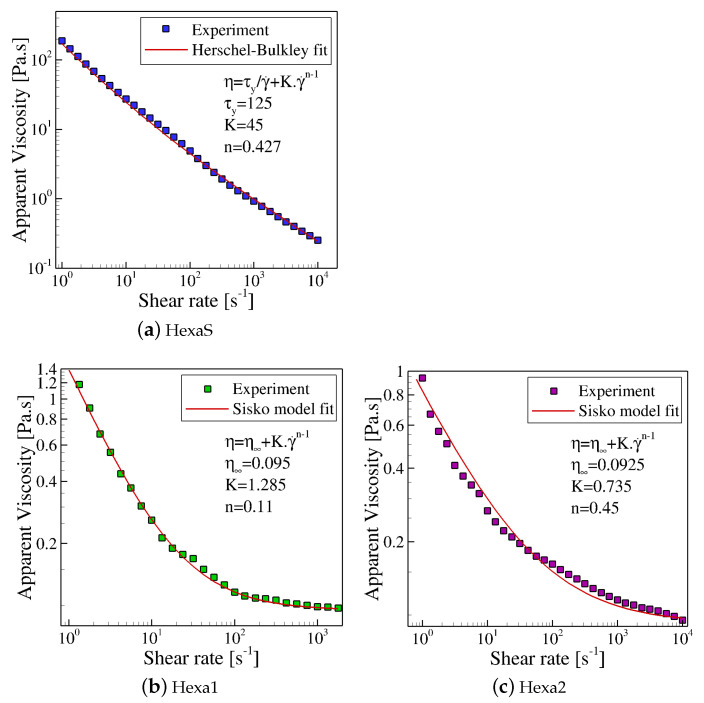
Graphene ink viscosity curves fitted with the GNF models: (**a**) HexaS fitted with the Herschel-Bulkley model; (**b**) Hexa-1 and (**c**) Hexa-2 fitted with the Sisko model.

Observing Figure 3a, it becomes obvious that Hexa-S samples exhibit a shear-thinning behavior so that the viscosity follows a power-law dependence with the shear rate and also a yield stress similar to other yield stress fluids, such as Kaolin suspensions [39], magnetorheological fluids [34], electrorheological fluids [31,33], and water/oil emulsions [40,41]. Based on that information, we selected the Herschel-Bulkley model for the ink Hexa-S, which is given by Equation (4):

(4) $$\tau =\tau_{y}+K\cdot {\dot{\gamma }}^{n},\mathrm{if}\;\tau \geq \tau_{y},$$

where $\tau_{y}$ is the yield stress parameter, *K* is the consistency parameter, and *n* is the flow index. If the applied stress is smaller than the yield stress value, the ink will not flow and it would behave like a solid. The viscosity function associated with the Herschel-Bulkley stress diverges to infinity as the strain rate approaches zero, e.g., start-up flow (Equation (5)):

(5) $$\lim_{\dot{\gamma }\to 0}\eta =\lim_{\dot{\gamma }\to 0}\frac{\tau_{y}}{\dot{\gamma }}+K\cdot {\dot{\gamma }}^{n-1}=\infty .$$

This divergence makes it difficult for the model to converge numerical simulations; in order to overcome this situation, it is common to implement an artificial upper-bounding viscosity ($\eta_{0}=125$ Pa·s). Thus, the Herschel-Bulkley fluid can be approximated as a GNF model with a dynamic viscosity given as follows:

(6) $$\eta =\left\{\begin{array}{cc} \frac{\tau_{y}}{\dot{\gamma }}+K\cdot {\dot{\gamma }}^{n-1}, & \mathrm{if}\;\tau \geq \tau_{y}\\ \eta_{0}, & \mathrm{if}\;\tau <\tau_{y} \end{array}\right.$$

The Herschel-Bulkley model is already available in the OpenFOAM computational library [42]. Figure 3a shows experimental data fitted with the Herschel-Bulkley model, and the values obtained for the different parameters are shown in Equation (4).

Regarding the Hexa-1 and Hexa-2 inks, their viscosity curves (Figure 3b,c) exhibited shear thinning behavior following a power-law dependency with the shear rate until reaching a plateau at high shear rates. The best fit for this behavior of the experimental data is given by the Sisko model [38], which has the following expression for the viscosity function:

(7) $$\eta =\eta_{\infty }+K\cdot {\dot{\gamma }}^{n-1},$$

where $\eta_{\infty }$ is the asymptotic plateau value of the viscosity at high shear rates, *K* is the consistency parameter, and *n* is the flow index.

The Sisko model has already been implemented into the open-source OpenFOAM computational library by Fernandes et al. [43]. Figure 3b,c show the experimental data fitted with the Sisko model and the values obtained for the different parameters in Equation (7) for the Hexa-1 and Hexa-2 inks.

#### 3.2.2. Surface Tension

The volume of fluid (VOF) technique was considered to capture the interface between the fluids (here air and liquid). The VOF interface capturing method was first proposed by Hirt and Nichols [44], and it uses a scalar function α, the so-called volume fraction, to define if the domain region that is occupied by liquid (α=1), if it is empty of that liquid (α=0), or, ultimately, if it corresponds to an interface between different liquids (0<α<1). Therefore, Equations (1) and (2) will be solved in combination with the transport equation for volume fraction (Equation (8)):

(8) $$\frac{\partial \alpha }{\partial t}+\nabla \cdot \left(u\alpha \right)=0,$$

in which α varies in the range of 0≤α≤1. In the VOF method described by Hirt and Nichols [44], the transport equation for an indicator function given by Equation (8) is solved, and, in the momentum equations, the surface tension effects at the interface are considered (Equation (2)). The surface tension at the interface generates an additional pressure gradient, resulting in a force, which is evaluated per unit volume using the continuum surface force (CSF) model [45]. The surface tension force, $f_{s}$, is calculated as follows [45,46]:

(9) $$f_{s}=\sigma \left(\nabla \cdot \left(\frac{\nabla \alpha }{\left|\nabla \alpha \right|}\right)\right)\left(\nabla \alpha \right),$$

where σ is the surface tension coefficient, ∇α=n is the vector normal to the interface [45], and the term in the middle is the mean curvature of the free surface. The fluid properties, which are density and dynamic viscosity, can also be obtained at each computational cell using the average volume fraction [46]

(10) $$\rho =\alpha \rho_{l}+\left(1-\alpha \right)\rho_{a},$$

(11) $$\eta =\alpha \eta_{l}+\left(1-\alpha \right)\eta_{a},$$

where indexes *l* and *a* are representative of liquid and air, respectively.

### 3.3. Numerical Method

The OpenFOAM 2.4.0 framework is used to numerically solve the fluid flows between the gravure cell and the substrate. This framework has previously been validated for the simulation of multiphase flows [47,48], and it also provides the capability of dynamic mesh handling [49,50]. These two features make the framework ideal for the numerical simulations of this transient free-surface fluid-flow problem, where the fluid-air interface changes its shape and position as long as the gravure cell separates from the substrate. Among OpenFOAM’s solvers, multiphaseInterDyMFoam was chosen to impose the motion of the gravure cell using the dynamic mesh feature and to solve the governing equations for a multiphase flow (i.e., two or more fluid phases).

For the numerical discretization of the governing equations presented in Section 3.2, the cell-center finite volume method is used. The coupling between the pressure and velocity fields is achieved through the segregated PIMPLE algorithm, which is a combination of the PISO (Pressure Implicit with Splitting of Operators) and SIMPLE (Semi-Implicit Method for Pressure-Linked Equations) algorithms [46,51]. The transient and source terms are discretized using the midpoint rule and integrated over cell volumes. The implicit Euler scheme is used for the discretization of the time derivative terms. The spatial derivative terms (diffusion and convective terms) are converted into surface integrals bounding each cell, making use of Gauss’s theorem. The cell face values are obtained by interpolation and then summed up to obtain the surface integral. For the evaluation of gradients, a linear face interpolation is used [46]. Additionally, to improve the efficiency of the numerical calculations, the OpenFOAM framework is capable of performinging adaptive time step control without losing the stability of the solution procedure. The time step is adjusted at the beginning of the time iteration loop based on the Courant number, which is defined as

(12) $$\begin{array}{ccc} Co & = & \frac{|u_{f}\cdot S_{f}|}{d\cdot S_{f}}\Delta t, \end{array}$$

where $u_{f}$ is the velocity of the fluid cell, $S_{f}$ is the vector of the surface of the cell pointing towards the direction of mass flux, d is a vector joining the control volume centroids sharing the same face, and Δt is the time step. Using values for $u_{f}$ and Δt from a previous time step, a maximum local Courant number, $Co^{0}$, is calculated, and the new time step is given by

(13) $$\begin{array}{ccc} \Delta t^{n} & = & \min\left\{\frac{Co_{max}}{Co^{0}}\Delta t^{0},\left(1+\lambda_{1}\frac{Co_{max}}{Co^{0}}\right)\Delta t^{0},\lambda_{2}\Delta t^{0},\Delta t_{max}\right\}, \end{array}$$

where $\Delta t_{max}$ and $Co_{max}$ are user-defined limit values for the time step and Courant number, respectively. The increase of the time step is damped using factors $\lambda_{1}$ and $\lambda_{2}$ [52]. During the first runs, it was found that the limit value for the Courant number should not exceed $Co_{max}=0.5$, which was also the value that was used for all the simulations performed in this study. The size of the time steps varied between values on the order of $10^{-5}$ s to $10^{-4}$ s during the calculations.

Additionally, the OpenFOAM framework solves the phase fraction equation in several subcycles, $n_{sc}$ (in this work equal to 1, which was found to be sufficient to obtain accurate results), within a single time step, Δt. Thus, the time step to be used in a single time subcycle is computed as:

(14) $$\begin{array}{ccc} \Delta t_{sc} & = & \frac{\Delta t}{n_{sc}}. \end{array}$$

In each subcycle, the phase fraction α is computed, and the corresponding mass flux, $F_{sc,i}$, is obtained through the cell faces. Afterwards, the total mass flux, *F*, of the global time step, which is needed in the momentum balance equations, is computed with the following equation:

(15) $$\begin{array}{ccc} F & = & \rho u_{f}\cdot S_{f}=\sum_{i=1}^{n_{sc}}\frac{\Delta t_{sc}}{\Delta t}F_{sc,i}. \end{array}$$

With this algorithm, a more accurate solution of the phase fraction equation is obtained, enabling, at the same time, the use of a greater global time step for the solution of other transport equations.

#### 3.3.1. Mesh

**Figure 4:**
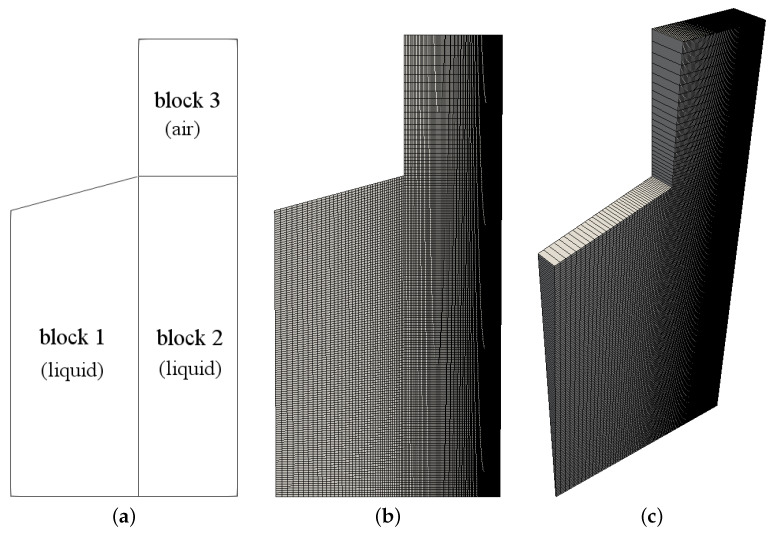
Mesh employed in the numerical calculations of the gravure cell printing process: (**a**) the blocks based on which the domain and the mesh have been created, (**b**) front view, and (**c**) perspective view of the axisymmetric wedge domain.

The domain shown in Figure 2 is spatially discretized using a structured mesh, which is generated using the blockMesh utility. The mesh is created using three blocks as shown in Figure 4a; two of them consist of liquid (block 1 and block 2), and the other block contains air (block 3). In the liquid zone, 40 cells are covering the horizontal length of block 1, while the horizontal length of block 2 is covered by 120 cells. A stretch is imposed in both blocks 1 and 2 with the minimum to maximum cell height aspect ratio equal to 0.25 in order to have a higher resolution near the substrate. Radially, 130 uniform cells cover the liquid zone in blocks 1 and 2. In block 3, a stretched mesh is generated with 24 cells radially covering the air zone, with the minimum to maximum cell height aspect ratio equal to 0.25 to have a finer grid at the liquid-air interface (see Figure 4).

For the mesh motion, the velocity U is given to the gravure cell to move away from the substrate, and the mesh boundary at substrate has a uniform constant velocity equal to zero. At the top, a slip boundary condition is imposed.

The multiphaseInterDyMFoam solver is employed to handle dynamic meshes for solving the governing equations of the two-phase flow. This solver has been recently explained, used, and validated by Fakhari and Galindo-Rosales [53].

#### 3.3.2. Validation of the Numerical Method

**Figure 5:**
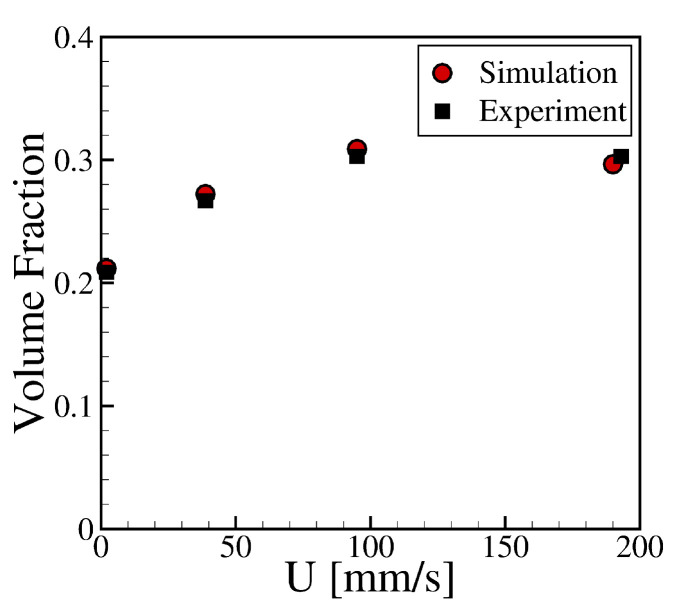
Comparison between the experimental and numerical results of the volume transferred to the substrate at different separation velocities, U, obtained for an aqueous solution of 20 K PEO at 20 wt%. The experimental data were extracted from the work of Sankaran and Rothstein [20].

To validate the numerical simulations, we numerically replicated the experiments developed by Sankaran and Rothstein [20] for a Newtonian fluid consisting of an aqueous solution of 20 K polyethylene oxide (PEO) at 20 wt% PEO. The simulations considered an initial cylindrical fluid held between the substrate and the cell, with a separation $L_{0}$ providing an aspect ratio of $L_{0}/R=0.3$. The gravure cell was then separated at a constant velocity normal from the substrate. A liquid bridge was formed between the substrate and the gravure cell, which progressively decreased in radius and eventually broke. The speed was varied from 2 mm/s ≤U≤190 mm/s. The percentage of fluid volume transferred to the substrate was calculated and compared with the experimental results provided by Sankaran and Rothstein [20]. As can be observed in Figure 5, there is an excellent agreement between the volume fraction measured experimentally and the numerical prediction.

#### 3.3.3. Validation of the Constitutive Models

**Figure 6:**
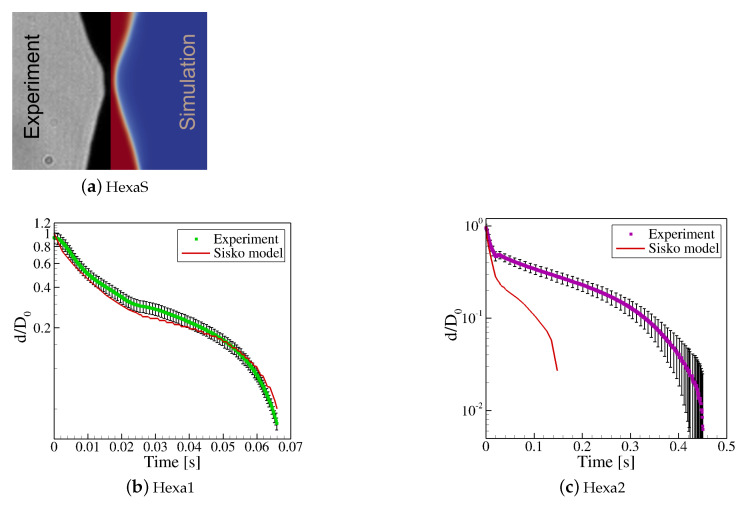
Comparison between the numerical predictions of the constitutive models and the experimental results obtained in the CaBER for the three inks: (**a**) HexaS, (**b**) Hexa1, and (**c**) Hexa2.

The accuracy of the predictions obtained by the numerical algorithm when using the two GNF models presented above was assessed by comparing the predictions to the experimental data obtained in the CaBER rheometer. Figure 6a illustrates the agreement between the filament profile recorded with the high-speed camera and the filament profile predicted by the Herschel-Bulkley model (Equation (6)). Figure 6b,c display the time evolution of the minimum normalized diameter obtained experimentally for Hexa1 and Hexa2 and the predictions made by the Sisko model (Equation (7)). It can be observed that in the case of Hexa1, the ink is clearly inelastic, as the model is able to capture the filament thinning process; however, in the case of Hexa2, the ink exhibits its viscoelastic nature (inset pictures [54]). The internal elastic stresses make the filament live longer, and the Sisko model is unable to follow that evolution. Therefore, in the subsequent analysis of the numerical results of the gravure printing process, we consider only the predictions obtained by the numerical code when using the HexaS and the Hexa1 samples. A study of the influence of the elasticity and length scale in the gravure printing process of the Hexa2 sample deserves a full paper on its own, which is currently being developed.

## 4. Results and Discussion

### 4.1. Analysis of the Flow Kinematics

**Figure 7:**
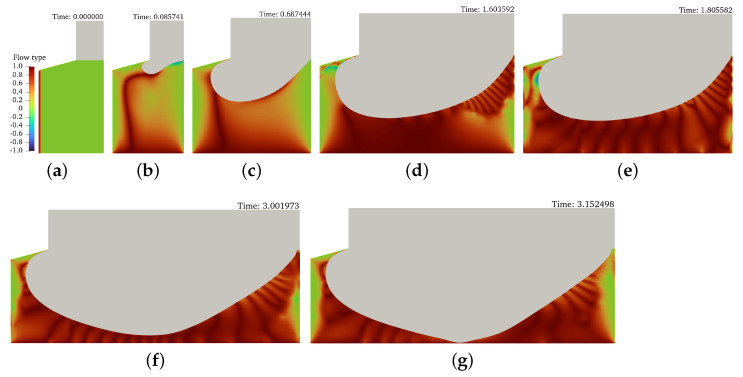
(**a**–**g**) The contour plots of flow type at different time steps for the simulation of HexaS ink and the gravure velocity U = 2 mm/s.

**Figure 8:**
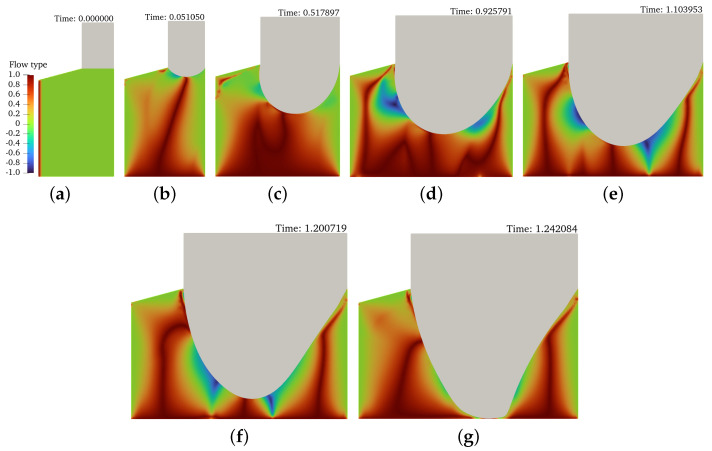
(**a**–**g**) The contour plots of flow type at different time steps for the simulation of Hexa-1 ink and the gravure velocity U = 2 mm/s.

**Figure 9:**
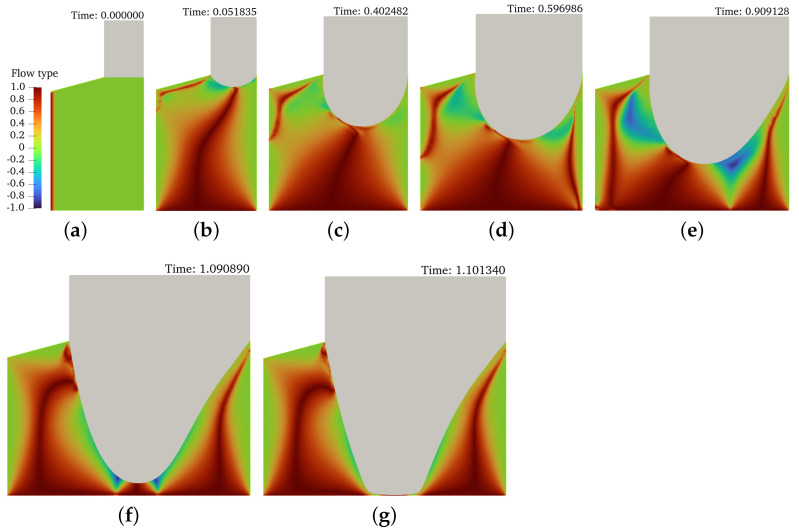
(**a**–**g**) The contour plots of flow type at different time steps for the simulation of Newtonian ink and the gravure velocity U = 2 mm/s.

The gravure printing process imposes complex flow kinematics to the liquid inks. The complexity of the flow can be clearly represented by the flow-type parameter [55], defined as

(16) $$\xi =\frac{∥D∥-∥\Omega ∥}{∥D∥+∥\Omega ∥},$$

where $∥D∥=\sqrt{\frac{D:D^{T}}{2}}$ is the magnitude of the rate-of-deformation tensor $D=\frac{1}{2}\left(\nabla u+\nabla u^{T}\right)$, and $∥\Omega ∥=\sqrt{\frac{\Omega :\Omega^{T}}{2}}$ is the magnitude of the vorticity tensor $\Omega =\frac{1}{2}\left(\nabla u-\nabla u^{T}\right)$. Regardless of the velocity and size of the geometry of the gravure cell, it can be observed in Figure 7, Figure 8 and Figure 9 that a complex flow pattern evolves with time as well as where there are zones in which the flow is purely simple shear (ξ=0), another region in the flow domain where the flow is purely elongational (ξ=1), another part where the flow approaches a solid-body rotation (ξ=−1), and another portion showing a combination of them. From early times until the moment the liquid bridge breaks, for any of the considered inks, the fluid close to the end walls undergoes pure shear, whereas the fluid located in the axis of symmetry experiences uniaxial extension. The rheology of the ink seems to play a role in the flow patterns observed for the rest of the volume of the liquid filament. In the case of HexaS, rotation can be barely seen, whereas for Hexa1 and the Newtonian cases, rotation can be observed at the ink-air interface (Figure 8 and Figure 9). This can be justified based on the fact that HexaS shows yield stress and large viscosity values at low shear rates. Thus, it is considered as a very ductile soft solid; this high viscosity drop at the liquid-air interface prevents the rotation of the ink. However, in the Hexa1 and Newtonian inks, the viscosity gradient at the interface is much smaller, and, at the centre of the interface, the fluid undergoes pure extension but is surrounded by rotation, which travels inwards to reach the axis of symmetry, and when it reaches it, the liquid bridge breaks up. The presence of stripes in the flow-type contour plots for the HexaS sample observed in Figure 7 is quite remarkable; these stripes correspond to zones of pure extension, alternated by layers combining shear and extension, and connect the interface with the axis of symmetry. These shear bands resemble those observations in highly ductile solid materials under mechanical tensile tests during the onset of plasticity, and they are known as Luders bands [56]; thus, by analogy, these stripes observed in Figure 7 can be associated with the plastic deformation of the HexaS ink. That is when the yield stress is overcome and it starts to flow as a viscous liquid.

Figure 7, Figure 8 and Figure 9 are also very useful because they allow the observation of the influence of the rheology on the silhouette of the liquid bridge. Looking at the profile of the liquid bridge, it becomes evident that the HexaS sample shows the typical breakup profile for yield stress fluids, with the two end tips (Figure 7) [33,34,40,57]. On the contrary, Hexa1 necks as a power-law-like profile but is ultimately cut-off by the background Newtonian viscosity ($\eta_{\infty }$), and, in this case, the midpoint radius ultimately goes to zero linearly in time (Figure 8) [54]. Finally, the Newtonian case breaks, showing the typical pinch-off (Figure 9) [54].

### 4.2. Printing Map

**Figure 10:**
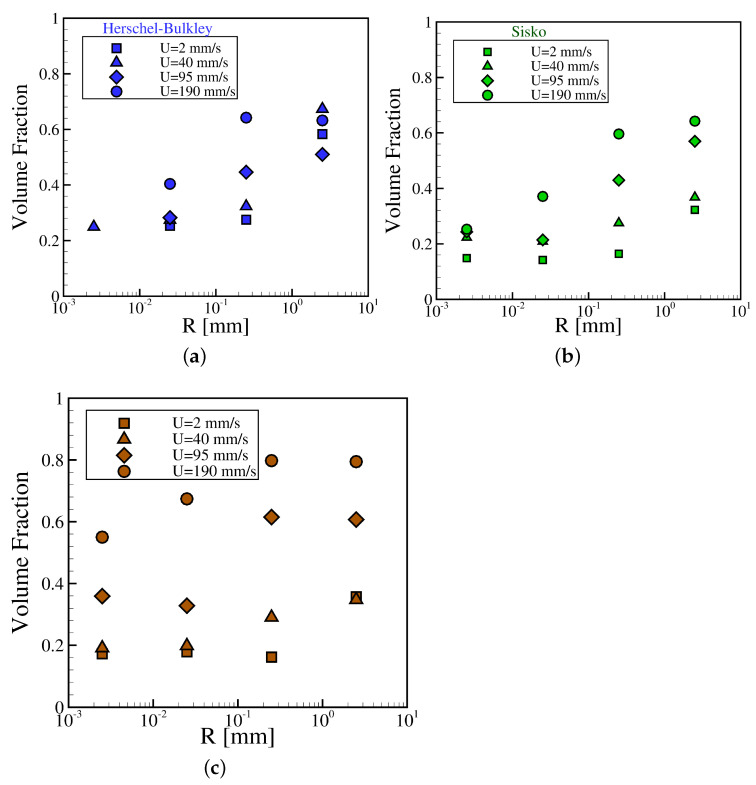
The volume fraction of different inks, i.e., (**a**) HexaS, (**b**) Hexa1, and (**c**) Newtonian, versus the outer radius of the gravure at different imposed velocities.

Once it is known how the rheology of the ink affects the kinematics of its flow during the gravure printing process, it is time to look at the effects on the practical aspects of the process, such as the volume fraction of ink transferred to the flat surface from the gravure cell. Figure 10 shows the dependence of the volume fraction of different inks, i.e., HexaS, Hexa1, and Newtonian, with the lengthscale of the gravure cell at different gravure velocities. It can be observed that for all the samples, at every length scale, the larger the velocity the higher the value of the volume fraction; whereas for a given gravure velocity, reducing the length scale diminishes the value of the volume fraction. Moreover, the largest values of volume fraction are achieved by the Newtonian ink at all lengthscales and for the largest gravure cell velocity. Plots for HexaS and Hexa1 show very similar trends when comparing them (Figure 10a,b); reducing the length scale below 250 μm results in a severe drop of the volume fraction, which worsens by diminishing the velocity of the gravure cell. HexaS exhibits higher values of volume fraction at the lowest velocities. Thus, we can conclude that shear thinning behavior is responsible for a diminishing in the transference of ink from the gravure cell to the flat surface when scaling down the size of the gravure cell. Therefore, it would be recommendable to formulate low-viscosity Newtonian inks in order to maximize the volume transfer at small length scales; moreover, using high velocities would also increase the volume fraction, apart from increasing the production of printed material.

As we can see from the results shown above, understanding and predicting the amount of graphene ink transferred by means of gravure printing is complex because different forces are involved, i.e., capillarity, viscosity, inertia, and gravity. Thus, a good approach for being able to predict the volume fraction transferred is through the tools of dimensional analysis. The dominant balance of the forces controlling the dynamics of the gravure printing process depends on the relative magnitudes of each underlying physical effect entering the set of governing equations. Using the Buckingham Pi theorem [58,59], the number of variables was reduced from nine ($L_{0}$, *R*, $R^{*}=H\tan\alpha$, σ, ρ, η, U, *g* and *P*) to six dimensionless groups:

- The Reynolds number: $Re=\frac{R\rho U}{\eta }$, where η is the dynamic viscosity of liquid sample, which has been determined from the constitutive equation of each ink, given the process strain rate defined as $\dot{\gamma }=\frac{U}{R_{0}}{\left(\frac{R_{0}}{L_{0}}\right)}^{-1/3}$ [22];
- The Weber number: $We=\frac{\rho RU^{2}}{\sigma }$, where ρ is the fluid density, σ is the surface tension of the air/sample interface, and *V* is the velocity of the gravure cell;
- The Froude number: $Fr=\frac{U}{\sqrt{gR}}$, where g=9.81 m/s${}^{2}$ is the gravitational constant, which can be converted into the Bond number as $Bo=\frac{We}{Fr^{2}}=\frac{\rho gR^{2}}{\sigma }$, relating the gravitational and capillary forces;
- The Euler number: $Eu=\frac{\Delta P}{\rho U^{2}}$, relating the local pressure difference and the kinetic energy per volume of the flow;
- The aspect ratio $\Lambda_{0}=\frac{L_{0}}{R}$;
- The gravure ratio $G_{R}=\frac{H\;\tan\;\alpha }{R}$.

In this study, the aspect ratio and gravure ratio were kept constant for all the cases; that is, neither the influence of the initial slenderness of the liquid bridge nor the shape of the gravure were considered in this study. McKinley [60] stated that the free surface flows of inelastic fluids can be characterized by the magnitude of the Reynolds number and the capillary number, which can be defined as the ratio between the Weber and the Reynolds numbers (Equation (17)):

(17) $$Ca=\frac{We}{Re}=\frac{\eta U}{\sigma },$$

and represents the relative importance of viscous drag forces and surface tension forces acting across an interface defined between the ink and the surrounding air.

**Figure 11:**
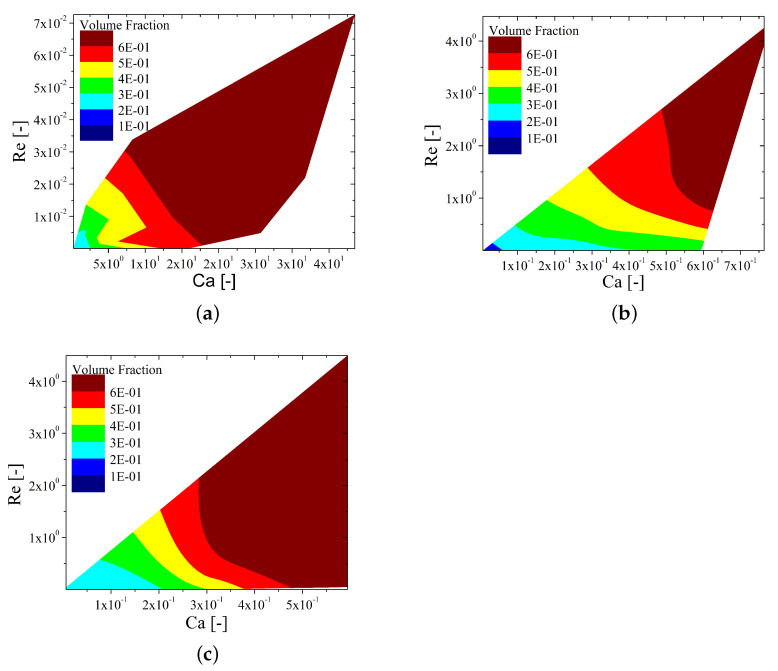
Contour plots of the volume fraction as a function of the capillary number and the Reynolds number for the three different inks considered: (**a**) HexaS, (**b**) Hexa1, and (**c**) Newtonian.

Figure 11 represents a contour plot of the volume fraction as a function of Re and Ca for all the cases considered in this study, i.e., the three inks, the four gravure velocities, and the four length scales. Depending on the intrinsic properties of the inks, a given gravure size and velocity result in different values of Re and Ca for the different inks, and, subsequently, the ranges in the axis of these contour plots are also different. HexaS exhibits the largest viscosity values and, consequently, the smallest Re ranges; whereas the Hexa1 and Newtonian inks, as their viscosities are on the same order of magnitude, have similar ranges of Re. Regarding Ca, the situation is opposite, i.e., HexaS has a larger range of capillary numbers than Hexa1 and the Newtonian ink. For the sake of a better comparison, the same range of values of volume fraction was considered for the three inks. In general terms, Figure 11 supports the discussion made above in Section 4.1, which states that the Newtonian ink is able to provide a larger transference of volume fraction for a wider range of printing considerations; moreover, in general terms, the larger the inertia and the lower the importance of the surface tension against the viscous drag, the larger the volume fraction transferred to the flat substrate will be, and vice versa. Thus, these plots allow for controlling the amount of ink deposited by controlling the dimensions and the velocity of the gravure cell without modifying the rheology of the ink.

**Figure 12:**
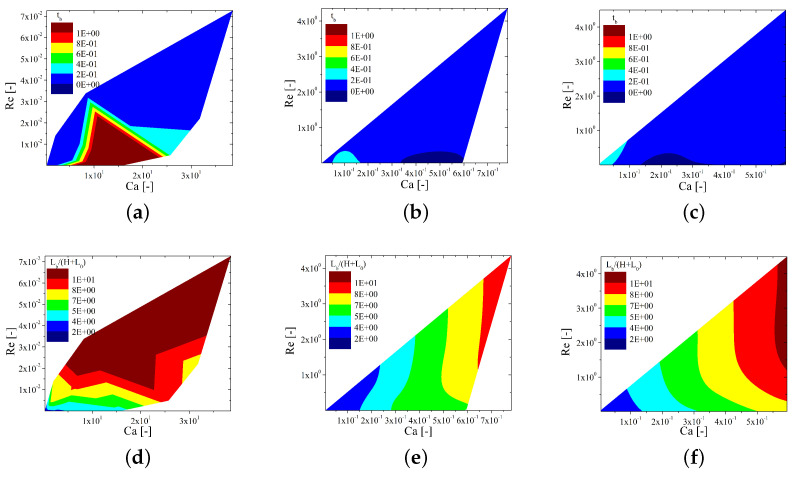
Contour plots of the breakup time (top row) and normalized breakup height (bottom row) as a function of the capillary and Reynolds numbers for the three different inks considered: HexaS (**a**,**d**), Hexa1 (**b**,**e**), and Newtonian (**c**,**f**).

The normalized length of the liquid bridge at the moment of the breakup and the breakup time have also been determined, each as a function of Re and Ca. The results are shown in Figure 12. HexaS gives both the largest breakup times and the longest filaments among the three inks for a wider range of Re and Ca values. This is mainly a result of its large viscosity values compared with the Hexa1 and Newtonian inks. These two latter inks show a normalized breakup length that grows with Ca and Re, but shear thinning behavior promotes shorter values of $\frac{L_{b}}{H+L_{0}}$; however, regarding the breakup time, shear thinning has no significant effect.

## 5. Summary and Remarks

In this study we characterized the rheological properties of three commercial graphene inks, which were not formulated with rheology modifiers to particularly fit the requirements of gravure printing technique but to provide excellent electromagnetic performance through customizable electrical resistance and wave attenuation levels. These inks were characterized rheologically under simple shear and uniaxial extensional flow, and that experimental information revealed that two of the inks could be considered inelastic (HexaS and Hexa1), whereas the third one (Hexa2) was viscoelastic. As this study aims at the analysis of the influence of shear thinning in the transferring process of gravure printing, we focused the attention on the inelastic graphene inks, leaving the effect of the elasticity for a future work. Thus, two generalized Newtonian fluid (GNF) models were fitted to the rheological data of the inks, i.e., the Herschel-Bulkley model for HexaS and the Sisko model for Hexa1, and they were able to replicate both experimental datasets under simple shear and elongational flow. The gravure printing process was modeled as in the work of Rothstein and co-workers [20,21,22] so that we focused our attention to the transference of fluid from the gravure cell to the flat substrate at different length scales from millimeters to micrometers. The numerical model was validated with their experimental data. Our numerical results allowed us to analyze the flow kinematics for the two inelastic inks, showing that, in the case of HexaS, the yield stress and large viscosity values inhibited the rotation at the liquid-air interface while the liquid bridge was thinning as a soft solid material; whereas, in the case of the Hexa1 ink, rotation appeared at the interface as in the Newtonian case. In general, the percentage of fluid transferred from the gravure to the flat surface was larger at larger lengthscales and larger velocities for the gravure cell. In order to better understand the simultaneous contributions of the shear thinning and length scales on the volume fraction transferred, the results were summarized in terms of the Reynolds and the capillary numbers by means of contour plots. Surprisingly, Newtonian behavior provided larger areas in the Re−Ca map than shear-thinning inks with large values of volume fraction of transferred ink, and these areas were located at high values of Re and Ca. These maps allow the determination of precisely the right dimensions and velocities of the gravure cells for each ink in order to control the right amount of ink being transferred to the substrate. Other important parameters for the gravure printing process, such as the distance from the flat substrate at which the breakup of the liquid bridge takes place or the time it takes to breakup were also mapped in terms of Re and Ca. The less viscous the ink, the faster it breaks; whereas the more viscous the ink is, the larger the filament needs to be in order to break it.

Finally, we conclude that any graphene ink could be printed out with the gravure printing process. One would just need to determine the Re−Ca print maps, adjust the velocity of the gravure cell to the length scale in order to get the maximum volume fraction, the minimum time to break, and the shortest distance. Nevertheless, very small lengthscales limit the volume fraction due to the more relevant role of surface tension; this latter effect could be counterbalanced if the ink was a low-viscosity Newtonian fluid instead of exhibiting shear-thinning behavior.

## Figures and Tables

**Table 1:** Main properties of the commercial graphene inks considered in this study.

Graphene Inks ${}^{+}$	Carrier Fluid	Solid Conc.	Density	Surface Tension
		(wt %)	(kg/m${}^{3}$)	(mN/m)
HexaFunction-1	DMSO+THF	3	1150	30.4 ± 0.1
HexaFunction-2	water	2	1000	29.4 ± 0.1
HexaShield	Toluene+Xylene	5	899	29.0 ± 0.1
Provided by Graphenest S.A.				Measured

^+^ Hereafter the three samples will be referred as Hexa1, Hexa2, and HexaS, respectively.

**Table 2:** Values of the parameters of the preshear protocol imposed on each sample.

Graphene Ink	Preshear Rate	Time Preshearing	Rest Time
	(s${}^{-1}$)	(s)	(s)
Hexa1	100	10	1
Hexa2	100	10	1
HexaS	10	150	0

## Data Availability

Not applicable.

## References

[1] Singh V., Joung D., Zhai L., Das S., Khondaker S.I., Seal S. (2011). Graphene based materials: Past, present and future. Prog. Mater. Sci..

[2] Akinwande D., Petrone N., Hone J. (2014). Two-dimensional flexible nanoelectronics. Nat. Commun..

[3] Tran T.S., Dutta N.K., Choudhury N.R. (2018). Graphene inks for printed flexible electronics: Graphene dispersions, ink formulations, printing techniques and applications. Adv. Colloid Interface Sci..

[4] Editorial (2017). Re-inventing print. Nat. Nanotechnol..

[5] Ostfeld A., Deckman I., Gaikwad A., Lochner C., Arias A. (2008). Screen printed passive components for flexible power electronics. Sci. Rep..

[6] Cen J., Kitsomboonloha R., Subramanian V. (2014). Cell Filling in Gravure Printing for Printed Electronics. Langmuir.

[7] Berggren M., Nilsson D., Robinson N.D. (2007). Organic materials for printed electronics. Langmuir.

[8] Grubb P.M., Subbaraman H., Park S., Akinwande D., Chen R.T. (2017). Inkjet Printing of High Performance Transistors with Micron Order Chemically Set Gaps. Sci. Rep..

[9] Secor E.B. (2018). Principles of aerosol jet printing. Flex. Print. Electron..

[10] Park J.U., Hardy M., Kang S.J., Barton K., Adair K., Mukhopadhyay D.K., Lee C.Y., Strano M.S., Alleyne A.G., Georgiadis J.G. (2007). High-resolution electrohydrodynamic jet printing. Nat. Mater..

[11] Søndergaard R.R., Hösel M., Krebs F.C. (2013). Roll-to-Roll fabrication of large area functional organic materials. J. Polym. Sci. Part B: Polym. Phys..

[12] Grau G., Cen J., Kang H., Kitsomboonloha R., Scheideler W.J., Subramanian V. (2016). Gravure-printed electronics: Recent progress in tooling development, understanding of printing physics, and realization of printed devices. Flex. Print. Electron..

[13] Secor E.B., Lim S., Zhang H., Frisbie C.D., Francis L.F., Hersam M.C. (2014). Gravure Printing of Graphene for Large-area Flexible Electronics. Adv. Mater..

[14] Kitsomboonloha R., Morris S.J.S., Rong X., Subramanian V. (2012). Femtoliter-Scale Patterning by High-Speed, Highly Scaled Inverse Gravure Printing. Langmuir.

[15] Calvi S., Maita F., Rapisarda M., Fortunato G., Valletta A., Preziosi V., Cassinese A., Mariucci L. (2018). Gravure printed organic thin film transistors: Study on the ink printability improvement. Org. Electron..

[16] Wu J.T., Francis L.F., Carvalho M.S., Kumar S. (2020). Cavity filling with shear-thinning liquids. Phys. Rev. Fluids.

[17] Wu C., Tetik H., Cheng J., Ding W., Guo H., Tao X., Zhou N., Zi Y., Wu Z., Wu H. (2019). Electrohydrodynamic Jet Printing Driven by a Triboelectric Nanogenerator. Adv. Funct. Mater..

[18] Dodds S., Carvalho M.D.S., Kumar S. (2009). Stretching and slipping of liquid bridges near plates and cavities. Phys. Fluids.

[19] Dodds S., Carvalho M., Kumar S. (2011). Stretching liquid bridges with moving contact lines: The role of inertia. Phys. Fluids.

[20] Sankaran A.K., Rothstein J.P. (2012). Effect of viscoelasticity on liquid transfer during gravure printing. J. Non-Newton. Fluid Mech..

[21] Khandavalli S., Rothstein J.P. (2017). Ink transfer of non-Newtonian fluids from an idealized gravure cell: The effect of shear and extensional deformation. J. Non-Newton. Fluid Mech..

[22] Khandavalli S., Lee J.A., Pasquali M., Rothstein J.P. (2015). The effect of shear-thickening on liquid transfer from an idealized gravure cell. J. Non-Newton. Fluid Mech..

[23] Graphenest HexaShield Product Brochure. https://graphenest.com.

[24] Galindo-Rosales F.J., Moldenaers P., Vermant J. (2011). Assessment of the Dispersion Quality in Polymer Nanocomposites by Rheological Methods. Macromol. Mater. Eng..

[25] Phair J.W., Lundberg M., Kaiser A. (2009). Leveling and thixotropic characteristics of concentrated zirconia inks for screen-printing. Rheol. Acta.

[26] Dullaert K. (2005). Constitutive Equations for Thixotropic Dispersions. Ph.D. Thesis.

[27] Galindo-Rosales F., Rubio-Hernández F. (2010). Static and Dynamic Yield Stresses of Aerosil 200 Suspensions in Polypropylene Glycol. Appl. Rheol..

[28] Raghavan S.R., Khan S.A. (1995). Shear-induced microstructural changes in flocculated suspensions of fumed silica. J. Rheol..

[29] Rodd L.E., Scott T.P., Cooper-White J.J., McKinley G.H. (2005). Capillary Break-up Rheometry of Low-Viscosity Elastic Fluids. Appl. Rheol..

[30] Campo-Deaño L., Clasen C. (2010). The slow retraction method (SRM) for the determination of ultra-short relaxation times in capillary breakup extensional rheometry experiments. J. Non-Newton. Fluid Mech..

[31] García-Ortiz J.H., Sadek S.H., Galindo-Rosales F.J. (2019). Influence of the Polarity of the Electric Field on Electrorheometry. Appl. Sci..

[32] García-Ortiz J.H., Galindo-Rosales F.J. (2020). Extensional Magnetorheology as a Tool for Optimizing the Formulation of Ferrofluids in Oil-Spill Clean-Up Processes. Processes.

[33] Sadek S.H., Najafabadi H.H., Galindo-Rosales F.J. (2020). Capillary breakup extensional electrorheometry (CaBEER). J. Rheol..

[34] Sadek S.H., Najafabadi H.H., Galindo-Rosales F.J. (2020). Capillary breakup extensional magnetorheometry. J. Rheol..

[35] Rubio M., Vega E.J., Herrada M.A., Montanero J.M., Galindo-Rosales F.J. (2020). Breakup of an electrified viscoelastic liquid bridge. Phys. Rev. E.

[36] Nunes J.M., Galindo-Rosales F.J., Campo-Deaño L. (2021). Extensional Magnetorheology of Viscoelastic Human Blood Analogues Loaded with Magnetic Particles. Materials.

[37] Morrison F.A. (2001). Understanding Rheology.

[38] Barnes H.A., Hutton J.F., Walters K. (1989). An Introduction to Rheology.

[39] Geffrault A., Bessaies-Bey H., Roussel N., Coussot P. (2021). Extensional gravity-rheometry (EGR) for yield stress fluids. J. Rheol..

[40] Niedzwiedz K., Arnolds O., Willembacher N., Brummer R. (2009). How to Characterize Yield Stress Fluids with Capillary Breakup Extensional Rheometry (CaBER)?. Appl. Rheol..

[41] Niedzwiedz K., Buggisch H., Willembacher N. (2010). Extensional rheology of concentrated emulsions as probed by capillary breakup elongational rheometry (CaBER). Rheol. Acta.

[42] Pimenta F. RheoTool User Guide v.5. https://github.com/fppimenta/rheoTool/blob/master/doc/user_guide.pdf.

[43] Fernandes C., Ferrás L., Araujo M., Nóbrega J. (2018). Development length in planar channel flows of inelastic non-Newtonian fluids. J. Non-Newton. Fluid Mech..

[44] Hirt C., Nichols B. (1981). Volume of Fluid (VOF) Method for the Dynamics of Free Boundaries. J. Comput. Phys..

[45] Andersson P. (2010). Tutorial MultiphaseInterFoam for the damBreak4phase Case.

[46] OpenFOAMWiki. https://openfoamwiki.net/index.php/InterFoam#cite_note-2.

[47] Erwee M.W., Reynolds Q.G., Zietsman J.H. (2016). Comparison of 2D and 3D computational multiphase fluid flow models of oxygen lancing of pyrometallurgical furnace tap-holes. JOM.

[48] Erwee M., Reynolds Q., Zietsman J., Bezuidenhout D. (2019). Multiphase flow modelling of lancing of furnace tap-holes: Validation of multiphase flow simulated in OpenFOAM. J. S. Afr. Inst. Min. Metall..

[49] Jasak H. Dynamic Mesh Handling in OpenFOAM. Proceedings of the 47th AIAA Aerospace Sciences Meeting Including the New Horizons Forum and Aerospace Exposition.

[50] Jasak H., Tukovic Z. Dynamic Mesh Handling in OpenFOAM Applied to Fluid-Structure Interaction Simulations. Proceedings of the V European Conference on Computational Fluid Dynamics ECCOMAS CFD.

[51] Issa R.I. (1986). Solution of the implicitly discretised fluid flow equations by operator–splitting. J. Comput. Phys..

[52] Rusche H. (2002). Computational Fluid Dynamics of Dispersed Two-Phase Flows at High Phase Fractions. Ph.D. Thesis.

[53] Fakhari A., Galindo-Rosales F.J. (2021). Parametric analysis of the transient back extrusion flow to determine instantaneous viscosity. Phys. Fluids.

[54] Mckinley G.H. (2005). Visco-elasto-capillary thinning and break-up of complex fluids. Annual Rheology Reviews.

[55] Ortega-Casanova J., Jimenez-Canet M., Galindo-Rosales F. (2019). Numerical study of the heat and momentum transfer between a flat plate and an impinging jet of power law fluids. Int. J. Heat Mass Transf..

[56] Yoshida F., Kaneda Y., Yamamoto S. (2008). A plasticity model describing yield-point phenomena of steels and its application to FE simulation of temper rolling. Int. J. Plast..

[57] Andrade R.J.E., Jacob A.R., Galindo-Rosales F.J., Campo-Deaño L., Huang Q., Hassager O., Petekidis G. (2020). Dilatancy in dense suspensions of model hard-sphere-like colloids under shear and extensional flow. J. Rheol..

[58] Buckingham E. (1914). On Physically Similar Systems; Illustrations of the Use of Dimensional Equations. Phys. Rev..

[59] Houghton E., Carpenter P., Collicott S.H., Valentine D.T. (2017). Chapter 1—Basic Concepts and Definitions. Aerodynamics for Engineering Students.

[60] McKinley G.H. (2005). Dimensionless Groups for Understanding Free Surface Flows of Complex Fluids.

